# Isotopic composition and major ion concentrations of national and international bottled waters in Costa Rica

**DOI:** 10.1016/j.dib.2021.107277

**Published:** 2021-08-11

**Authors:** Ricardo Sánchez-Murillo, Germain Esquivel-Hernández, Christian Birkel, Lucía Ortega

**Affiliations:** a Stable Isotopes Research Group and Water Resources Management Laboratory, Universidad Nacional, Heredia 86-3000, CR, USA; b Water and Global Change Observatory, Department of Geography, Universidad de Costa Rica, San Jose 2060, CR, USA; c International Atomic Energy Agency, Isotope Hydrology Section, Vienna 1400, Austria

**Keywords:** Imported and national-based bottled waters, Water stable isotopes, Chemical compositions, Water sources, Recharge elevations, Traceability

## Abstract

Global bottled water consumption has largely increased (14.35 billion gallons in 2020) [1], [2], [3], [4], [5] during the last decade since consumers are demanding healthier and safer forms of rehydration. Bottled water sources are normally labeled as mountainous and pristine mineral springs (fed by rainfall and snow/glacier melting processes), deep groundwater wells or industrial purified water. The advent of numerous international and national-based bottled water brands has simultaneously raised a worldwide awareness related to the water source and chemical content traceability [6]. Here, we present the first database of stable isotope compositions and reported chemical concentrations from imported and national-based bottled waters in Costa Rica. In total, 45 bottled waters produced in Costa Rica and 31 imported from USA, Europe, Oceania, and other countries of Central America were analyzed for δ^18^O, δ^2^H, and *d*-excess. Chemical compositions were obtained from available bottle labels. National-based bottle waters ranged from -2.47‰ to -10.65‰ in δ^18^O and from -10.4‰ to -78.0‰ in δ^2^H, while *d*-excess varied from +4.2‰ up to +17.0‰. International bottle waters ranged between -2.21‰ and -11.03‰ in δ^18^O and from -11.3‰ up to -76.0‰ in δ^2^H, while *d*-excess varied from +5.0‰ up to +19.1‰. In Costa Rica, only 19% of the brands reported chemical parameters such as Na^+^, K^+^, Ca^+2^, Mg^+2^, F^−^, Cl^−^, NO_3_^−^, SO_4_^−2^, CO_3_^−2^, SiO_2_, dry residue, and pH; whereas 27% of the international products reported similar parameters. The absence of specific geographic coordinates or water source origin limited a spatial analysis to validate bottled water isotope compositions versus available isoscapes in Costa Rica [7]. This database highlights the potential and relevance of the use of water stable isotope compositions to improve the traceability of bottled water sources and the urgent need of more robust legislation in order to provide detailed information (i.e., water source, chemical composition, purification processes) to the final consumers.

## Specifications Table

**Table:** 

Subject	Analytical Chemistry, Environmental Chemistry, Hydrology, Water Resources Management.
Specific subject area	Stable isotope compositions in bottled waters
Type of data	Graphs and Tables
How data were acquired	Laser spectroscopy for water stable isotopes analysis with an IWA-45EP water analyzer (Los Gatos Research, Inc., California, USA).
Data format	Raw Analyzed
Parameters for data collection	Imported and national-based bottled waters were classified as natural mineral water, spring water, purified water and not specified waters. Chemical compositions were extracted from available bottle labels, the parameters included: Na^+^, K^+^, Ca^+2^, Mg^+2^, F^−^, Cl^−^, NO_3_^−^, SO_4_^−2^, CO_3_^−2^, SiO_2_, dry residue, and pH. National-based samples were coded from CR1 to CR45; while imported samples were coded from FW1 to FW31.
Description of data collection	Bottled waters were purchased across a broad range of commercial stores in Costa Rica, Honduras, Panamá, Guatemala, and El Salvador. Samples were classified as national-based (Costa Rica), imported from USA, Europe, Oceania, and other countries of Central America. All samples were sealed and refrigerated at 5 °C until analysis at the Stable Isotopes Research Group, Universidad Nacional (Heredia, Costa Rica).
Data source location	Institution: Universidad Nacional City/Town/Region: Heredia Country: Costa Rica Latitude and longitude (and GPS coordinates) for collected samples/data: 10.094533, −84.058700 Elevation: 1153m asl.
Data accessibility	Repository name: https://www.hydroshare.org/ Data identification number: 10.4211/hs.86225d08252747d5a78477c1f74eb158 Direct URL to data: https://www.hydroshare.org/resource/86225d08252747d5a78477c1f74eb158/
Related research article	Sánchez‐Murillo, R. and C. Birkel, C, Groundwater recharge mechanisms inferred from isoscapes in a complex tropical mountainous region. Geophysical Research Letters. 43 (2016) 5060–5069. https://doi.org/10.1002/2016GL068888

## Value of the Data

• Our information provides the first database of stable isotope compositions and reported (from bottled water labels) chemical concentrations from imported and national-based bottled waters in Costa Rica.
• Bottled water isotopic compositions may be used in concert with available isoscapes to assist with the traceability of water sources used in the industry of bottled waters across the Central America region.
• Our data revealed a large degree of inconsistency in reporting chemical compositions both in national-based and imported bottled waters.
• This database highlights the urgent need of more robust legislation for the bottled water industry worldwide in order to provide detailed information to the final consumers related-but not limited-to water sources (origin and geographic coordinates), chemical compositions (a complete spectrum of major ion contents), and clarity of the purification processes used.

## 1 Data Description

**Fig. 1:**
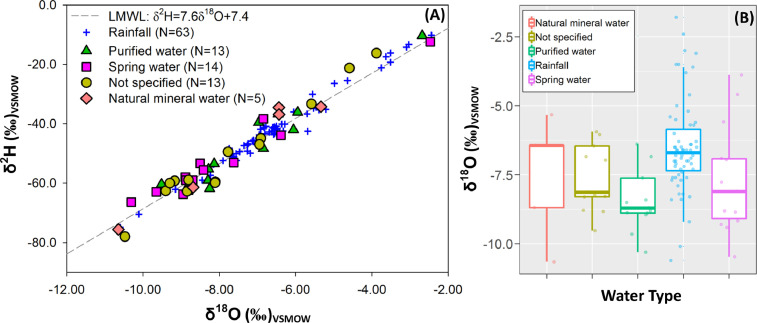
(A) Dual water isotope diagram including bottled waters classified as purified water (triangles), spring water (squares), natural mineral water (rhombi), and samples in which the water type appeared as not specified (circles). The LMWL of Costa Rica [8] was included as reference. Blue crosses denote the precipitation-weighted mean annual composition across 63 monitoring stations in Costa Rica [7,8]. (B) δ^18^O (in ‰) scattered/box plots per water type, including rainfall values of 63 monitoring stations in Costa Rica [7,8] as a reference. δ^18^O (in ‰) scattered/box plots include 25th, 75th, median, and outliers for each water type.

**Fig. 2:**
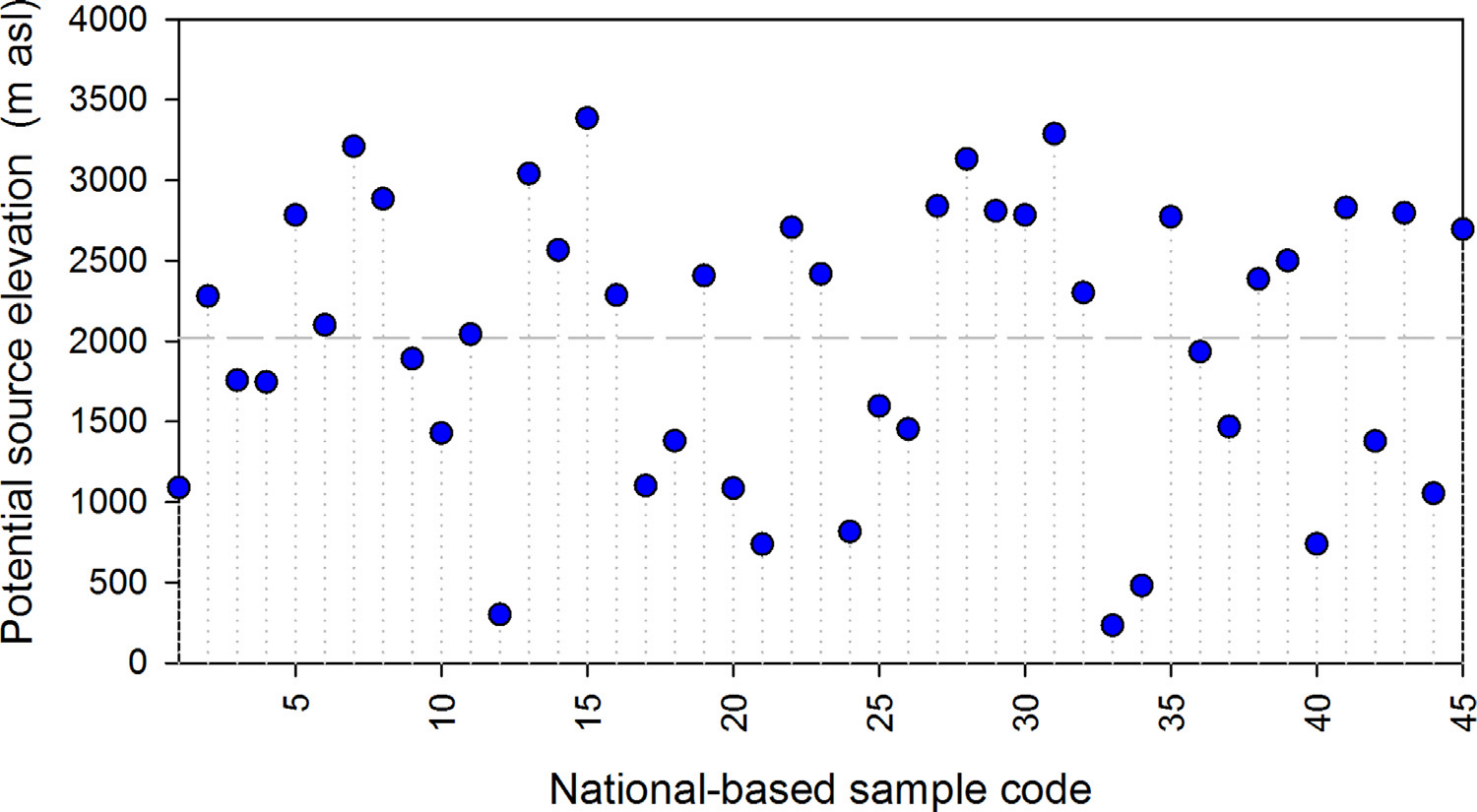
Potential elevation source (in m asl) for all national-based bottled waters in Costa Rica. The δ^18^O lapse rate (-1.4‰/km) was obtained from 63 monitoring stations in Costa Rica [7,8].

Fig. 1A shows a dual water isotope diagram including bottled waters classified as Purified Water, Spring Water, Natural Mineral Water, and samples in which the water type was Not Specified (i.e., unclear potential source). Overall, all samples exhibited a strong meteoric origin [7,8]. Based on available isoscapes of Costa Rica and recent monitoring efforts [7], [8], [9], only 4 bottled waters were in the range of typically enriched Caribbean-type water -2‰ to -4‰ in δ^18^O; Fig 1A), while 41 samples revealed Pacific-type δ^18^O compositions (ranging from -6 up to -11‰; Fig. 1B). Purified, Spring, and Not Specified bottled waters exhibited depleted δ^18^O values, confirming the strong bias and industry preference towards Pacific-type water sources [9]. Deuterium excess values ranged from + 4.2‰ up to + 17.0‰, with a mean value of + 11.0‰, which are in the range of rainfall across Costa Rica [7], [8], [9], [10], [11]. Potential source elevation (in m asl) were evaluated using an available isotopic lapse rate for Costa Rica derived from precipitation weighted mean annual δ^18^O compositions. Based on precipitation amounts, average annual weighted ratios were calculated across 63 monitoring stations [7,8]. The exisiting isotope monitoring network in precipitation provides a reliable spatial distibution across different climatic zones, elevation gradient, and biomes. For δ^18^O in rainfall, the altitude effect across the country averaged −1.4 ‰ per 1 km increased for stations above ∼340 m of elevation (*r^2^* = 0.43, *P* < 0.001). . In general, potential source elevations ranged from 233 up to 3386 m asl, with a mean value of 2020 m asl, which in turn reflects the large dependency of high elevation recharge processes and spring discharges for the bottled water industry in Costa Rica (Fig. 2).

**Fig. 3:**
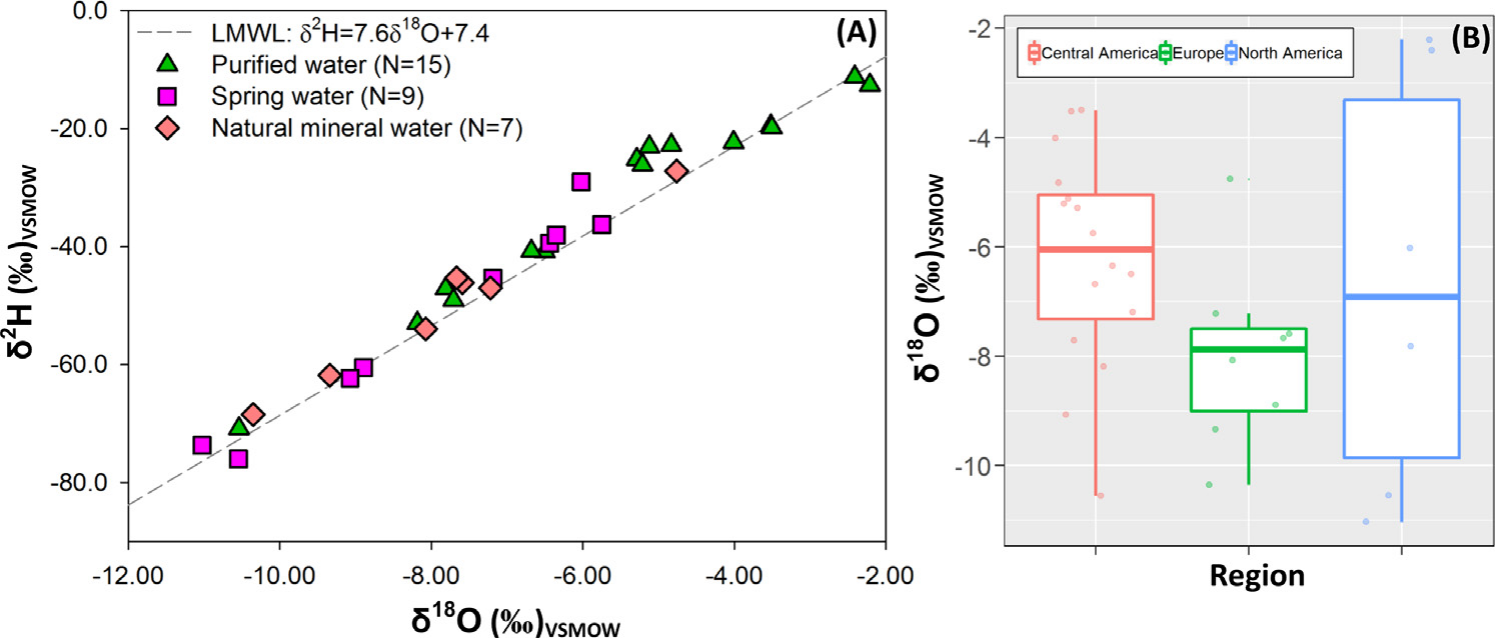
(A) Dual water isotope diagram including imported bottled waters classified as Purified Water (triangles), Spring Water (squares), and Natural Mineral Water (rhombi). The LMWL of Costa Rica [7,8] was included as reference. (B) δ^18^O (in ‰) scattered/box plots per region (one sample from Oceania was excluded). δ^18^O (in ‰) scattered/box plots include 25th, 75th, median, and outliers for each region.

Fig. 3A shows a dual water isotope diagram including imported bottled waters from Europe (Norway, Italy, Spain, and France), North America (USA and Mexico), Oceania (Fiji Islands), and Central America (Guatemala, El Salvador, Honduras, and Panama). In general, imported bottled waters covered a similar δ^18^O range (from -2‰ up to -12‰). Deuterium excess values of imported bottled waters varied from + 5.0‰ to + 19.1‰, with mean of + 12.4‰. As expected, bottled waters from the mountainous regions of Europe exhibited lower δ^18^O compositions due to strong latitudinal and temperature effects (Fig. 3B) [13,14]. The median δ^18^O composition of bottled waters from Central America corresponded to rainfall/groundwater isotope compositions in the Pacific domain of this region (Fig. 3B) [12].

**Fig. 4:**
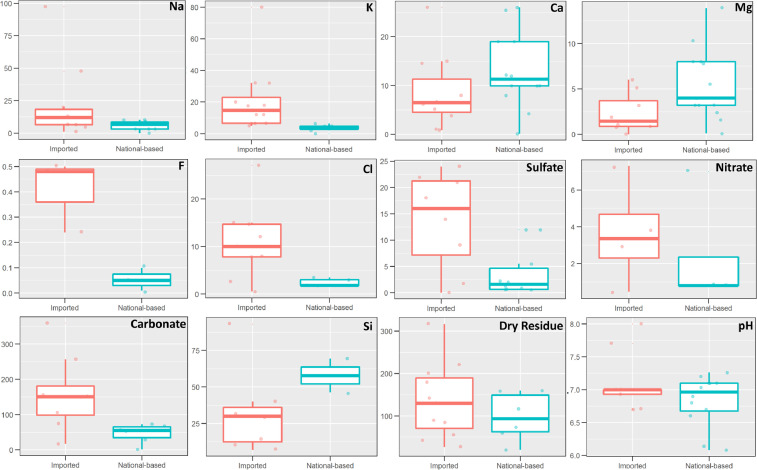
Reported chemical compositions of national-based and imported bottled waters in Costa Rica. All concentrations are expressed in mg/L (Si refers to SiO_2_). Scattered/box plots include 25th, 75th, median, and outliers for each group.

The traceability of chemical compositions in bottled waters has been a matter of debate worldwide [15], [16], [17], [18]. Fig. 4 shows major ion concentrations, dry residue, and pH reported in bottled water labels. In Costa Rica, only 19% of the brands reported a combination of chemical parameters such as Na^+^, K^+^, Ca^+2^, Mg^+2^, Fe^+2/+3^, F^−^, Cl^−^, NO_3_^−^, SO_4_^−2^, CO_3_^−2^, SiO_2_, dry residue, and pH; whereas 27% of the international products reported similar parameters (Fig. 4). Imported bottled waters reported greater concentrations in Na^+^, K^+^, F^−^, Cl^−^, NO_3_^−^, SO_4_^−2^, CO_3_^−2^, and dry residue. Major recharge/discharge processes in mountainous volcanic aquifers in Costa Rica are represented by high silicate and low pH values (Fig. 4). In general, there is a large degree of inconsistency in reporting chemical compositions both in national-based and imported bottled waters. However, it is important to highlight that each country has different acceptable limits of the chemical compositions in bottled waters.

## 2 Experimental design, materials and methods

### 2.1 Sample collection

National-based (*N* = 45) and imported (*N* = 31) bottled water samples were purchased between 2019–2020 across a broad range of commercial stores (all bottles were in perfect condition) in Costa Rica, Honduras, Panamá, Guatemala, and El Salvador. Samples were classified as national-based (Costa Rica), imported from USA, Europe, Oceania, and other countries of Central America. As this type of sampling is opportunistic, seasonal effects are not included in our analysis. All samples were sealed with parafilm and refrigerated at 5 °C until analysis at the Stable Isotopes Research Group, Universidad Nacional (Heredia, Costa Rica). Chemical compositions were extracted from available bottle labels, the parameters included: Na^+^, K^+^, Ca^+2^, Mg^+2^, Fe^+2/+3^, F^−^, Cl^−^, NO_3_^−^, SO_4_^−2^, CO_3_^−2^, SiO_2_, dry residue, and pH. National-based samples were coded from CR1 to CR45; while imported samples were coded from FW1 to FW31. All information is available at https://www.hydroshare.org/resource/86225d08252747d5a78477c1f74eb158/ [11]. CUASI´s hydrological repository Hydroshare (https://www.hydroshare.org/) is an online platform to share data, models, and code.

### 2.2 Stable Isotopes analysis

Samples were analyzed at the Stable Isotopes Research Group laboratory at the Universidad Nacional (Heredia, Costa Rica) using an IWA-45EP water analyzer (Los Gatos Research, Inc., California, USA) with a precision of ± 0.5‰ for δ^2^H and ± 0.1‰ for δ^18^O (1σ). Stable isotope compositions are expressed as δ^18^O or δ^2^H = (R_s_/R_std_ - 1)•1000, where R is the ^18^O/^16^O or ^2^H/^1^H ratio in a sample (s) or standard (std) and reported in the delta-notation (‰) relative to V-SMOW/SLAP scale. The instrument accuracy was assessed with a combination of in-house and primary international water standards (SMOW and SLAP). Deuterium excess was calculated as *d*-excess = δ^2^H - 8•δ^18^O [19].

## CRediT Author Statement

**Ricardo Sánchez-Murillo:** Conceptualization, Methodology, Formal analysis, Data curation, Writing – original draft; **Germain Esquivel-Hernández:** Writing – review & editing, Writing – original draft; **Christian Birkel:** Writing – review & editing, Writing – original draft; **Lucía Ortega:** Writing – review & editing, Writing – original draft.

## Declaration of Competing Interest

The authors declare that they have no known competing financial interests or personal relationships that have, or could be perceived to have, influenced the work reported in this article.
